# Mitochondrial heterogeneity: subpopulations with distinct metabolic activities

**DOI:** 10.1038/s41392-025-02130-0

**Published:** 2025-02-07

**Authors:** Fabian den Brave, Swadha Mishra, Thomas Becker

**Affiliations:** Institute of Biochemistry and Molecular Biology, Faculty of Medicine, University of Bonn, Bonn, Germany

**Keywords:** Biochemistry, Cell biology

In a recent study published in *Nature*, Ryu et al.^1^ demonstrate the presence of metabolically distinct mitochondrial subpopulations within one cell. One mitochondrial subpopulation contains the F_1_F_O_-ATP synthase for oxidative phosphorylation (OXPHOS), while a second population performs reductive biosynthesis of proline and ornithine. The separation of mitochondria into two functionally distinct pools is reversible and depends on their fusion and fission.

**Fig. 1:**
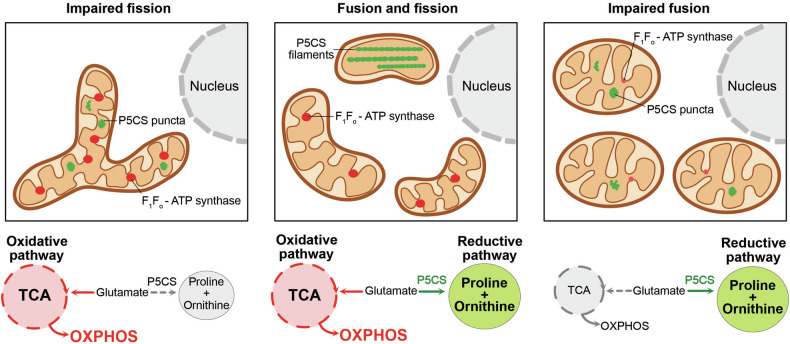
Mitochondrial fusion and fission enable formation of mitochondrial subcompartments. Middle panel: Under conditions of high oxidative phosphorylation (OXPHOS) distinct populations of mitochondria are formed dependent on mitochondrial fusion and fission. One mitochondrial subpopulation contains cristae and the F_1_F_O_-ATP synthase, whereas a second population lacks cristae and contains filaments of the enzyme delta-1-pyrroline-5 carboxylate synthase (P5CS) required for proline and ornithine synthesis. The presence of two distinct mitochondrial subpopulations allows conversion of glutamate to an intermediate of the tricarboxylic acid (TCA) cycle to power ATP production via OXPHOS and the metabolization of glutamate via P5CS for reductive biosynthesis of proline and ornithine in the same cell. Left panel: Impaired mitochondrial fission results in network expansion and prevents separation of F_1_F_O_-ATP synthase and P5CS into distinct mitochondrial populations. As a consequence, increased oxidative utilization of glutamate impairs the metabolization of glutamate for biosynthesis of proline and ornithine. Right panel: Impaired fusion prevents the exchange of mitochondrial content, resulting in fragmented mitochondria containing reduced levels of the F_1_F_O_-ATP synthase. In these mitochondria, OXPHOS activity is reduced, while biosynthesis of proline and ornithine is unaffected

Eukaryotic cells contain different cellular organelles that provide compartments to spatially separate processes including metabolic pathways. Mitochondria are multifunctional organelles important for energy production via oxidative phosphorylation (OXPHOS), synthesis of amino acids, lipids or heme and serve as cellular signaling hubs in apoptosis or innate immunity. Metabolic processes within mitochondria are often highly interconnected and share common intermediates. In order to understand how different metabolic pathways are interconnected in mitochondria, Ryu and colleagues used the STRING database that combines results of experimental data and computational analysis for prediction of protein-protein interactions to model the protein networks of mitochondrial enzymes. They defined three main clusters, the tricarboxylic acid (TCA) cycle, amino acid biosynthesis and one-carbon metabolism, and found that the delta-1-pyrroline-5 carboxylate synthase (P5CS) links all three clusters.^1^ P5CS performs the rate-limiting step in the biosynthesis of proline and ornithine, which is the reduction of glutamate to glutamate semialdehyde. Glutamate can also be deaminated to α-ketoglutarate, which is utilized in the TCA cycle to power ATP production via OXPHOS. Thus, the conversion of glutamate represents a critical branching point of mitochondrial metabolism, where the conversion of glutamate to an intermediate of the TCA cycle competes with the utilization of glutamate for reductive biosynthesis of amino acids. The authors determined the activity of these pathways under conditions of high or low OXPHOS activity in mouse embryonic fibroblasts. Interestingly, under conditions of increased glutamate utilization for OXPHOS, no reduction in the synthesis of proline and ornithine was observed. How does the cell carry out two competing reactions at the same time? Surprisingly, distinct subpopulations of mitochondria are present within the same cell under conditions with high OXPHOS activities such as growth on medium with galactose as the carbon source. Such conditions lead to the formation of enzymatic active P5CS-containing filaments, clustering the protein into few spots inside mitochondria. Immunofluorescence analysis revealed that mitochondria containing P5CS filaments were devoid of the F_1_F_O_-ATP synthase subunits, whereas mitochondria containing the F_1_F_O_-ATP synthase lack P5CS filaments. Analyzing the ultrastructure of these mitochondria by correlative light and electron microscopy revealed that P5CS-containing mitochondria are devoid of cristae membranes, whereas P5CS-negative mitochondria retained normal cristae. P5CS-containing mitochondria lack Mic60 and are impaired in the dimerization of the F_1_F_O_-ATP synthase, which both could contribute to the loss of cristae. Collectively, these experiments suggest the presence of two distinct subpopulations of mitochondria within the same cell that either display high OXPHOS activity or use glutamate for reductive proline and ornithine biosynthesis (Fig. 1).^1^

How are these mitochondrial subpopulations formed? The reversible formation of P5CS filaments appears to promote the segregation of this protein into distinct regions within mitochondria. The mitochondrial network is constantly remodeled by opposing fusion and fission events.^2^ In cells deficient in fusion, mitochondria were still able to produce proline, but displayed impaired OXPHOS activity (Fig. 1). Conversely, cells blocked in mitochondrial fission are impaired in the formation of the mitochondrial subpopulations and showed efficient OXPHOS, while their ability to synthesize proline was reduced (Fig. 1). These observations suggest a critical role of mitochondrial fusion and fission in establishing mitochondrial heterogeneity with functionally distinct subpopulations. In this scenario, P5CS forms filaments, which are then separated into mitochondrial subpopulations by fission. The data also reflect the importance of the functional separation of mitochondria into subpopulations to maintain full capacity to utilize glutamate for oxidative and reductive pathways in the cell (Fig. 1).

The separation of organelles into distinct subpopulations provides a new layer of complexity to organize and compartmentalize cellular functions. Mitochondrial heterogeneity has been described with respect to differences in morphology, ultrastructure, subcellular localization, membrane potential or mitochondrial DNA.^3^ For instance, the separation of mitochondria with depleted membrane potential or mitochondrial DNA mutations facilitates the clearance of damaged mitochondria by mitophagy.^3^ However, only little is known about the metabolic specialization of mitochondrial subpopulations. In addition to the presented study, distinct mitochondrial subpopulations with specific metabolic activities have been found in adipocytes.^4^ One mitochondrial subpopulation binds to lipid droplets, whereas a second one is present in the cytosol. The lipid droplet-associated mitochondria exhibit an increased OXPHOS activity to deliver ATP for lipid droplet expansion.^4^ Future studies have to reveal whether further mitochondrial subpopulations are formed to maintain the full metabolic capacity of the cell under different growth or stress conditions.

The functional heterogeneity of mitochondria raises interesting questions about the molecular mechanism separating and maintaining mitochondrial subpopulations. Fusion and fission processes are critical for the separation of mitochondrial pools. Their role in the establishment of mitochondrial heterogeneity may contribute to the multitude of defects observed in cells with impaired mitochondrial fusion and fission. The molecular mechanisms that govern segregation of P5CS into filaments or lead to the loss of the cristae remain unknown. Interestingly, systematic complexome profiling of yeast mitochondria revealed that mitochondrial proteins form a large number of protein assemblies.^5^ Formation of mitochondrial subpopulations would involve segregation of a subset of assembled protein machineries like the F_1_F_o_-ATP synthase into two distinct populations of mitochondria. The identification and functional characterization of the underlying molecular mechanism represent interesting challenges for future research. For instance, mitochondria depend on the co- and posttranslational import of more than thousand proteins in the cell. Therefore, maintenance of mitochondrial subpopulations depends on the import of different proteins. How protein targeting to and import into distinct mitochondrial subpopulations in the same cell is accomplished remains unclear. Furthermore, distinct proteases remove orphaned and aberrant proteins within mitochondria.^2^ Whether these proteases monitor formation of mitochondrial subpopulations remains to be established. In conclusion, the presence of mitochondrial heterogeneity suggests further complexity of coordination and regulation of established mitochondrial processes.
